# Evaluation of Bitemark Analysis’s Potential Application in Forensic Identification: A Systematic Review

**DOI:** 10.3390/diagnostics14111180

**Published:** 2024-06-04

**Authors:** Nikolaos Christoloukas, Anastasia Mitsea, Aliki Rontogianni, Evangelos Papadakis, Christos Angelopoulos

**Affiliations:** 1 Department of Oral Diagnosis and Radiology, School of Dentistry, National and Kapodistrian University of Athens, 11527 Athens, Greece; 2 Division of Dental Technology, Department of Biomedical Sciences, University of West Attica, 12243 Athens, Greece

**Keywords:** bitemark, human, forensic identification techniques

## Abstract

Bitemark analysis involves the examination of both patterned injuries and contextual circumstances, combining morphological and positional data. Considering the uniqueness of human dentition, bitemarks caused by teeth on skin or impressions on flexible surfaces could assist in human identification. Aims: to investigate the available literature systematically and evaluate the scientific evidence published over the past decade concerning the potential application of bitemark analysis in forensic identification. Methods: Two researchers meticulously searched electronic databases from January 2012 to December 2023, including Scopus, PubMed, Web of Science, and the Cochrane Library. Adhering to the PRISMA statement guidelines, this review employed appropriate medical subject headings (MeSHs) and free-text synonyms. Strict inclusion and exclusion criteria were applied during article retrieval. Results: The findings yielded controversial outcomes. Approximately two-thirds of the articles concluded that bitemark analysis is useful in forensic identification, while the remaining articles did not report statistically significant outcomes and cautioned against relying solely on bitemark analysis for identification. Conclusions: The authors assert that bitemark analysis can be a reliable and complementary method for forensic identification, contingent upon the establishment and adoption of a universally accepted global protocol for data collection, processing, and interpretation. Undoubtedly, recent years have witnessed a notable increase in research focused on bitemark identification, driven by the goal of achieving quantitative, objective, reproducible, and accurate results.

## 1. Introduction

Bitemark analysis maintains significant importance in forensic odontology, as it can wield substantial influence, whether within a legal framework or in evaluating the well-being of children considered to be at risk [1]. Bitemarks act as impressions created by teeth on the skin or other flexible surfaces [2,3]. Bitemark analysis involves examining both the patterned injury and the surrounding circumstances. This procedure is denoted as bitemark comparison in the event of comparing an injury to a suspect or a specific population group [4]. The accuracy of teeth impressions on the bitten material is essential for bitemark analysis, which relies on the uniqueness of human dentition [5]. Forensic odontologists conduct examinations, interpretations, analyses, and prepare reports regarding marks or bruises suspected to be tooth-related. Occasionally, they are subjected to cross-examination in a court of law [1]. The process of identifying an injury as a bitemark is intricate, involving numerous factors that must be considered. These factors encompass the location and dimensions of the injury, the skin’s flexibility and elasticity, the depth and composition of structures beneath the injured skin area, the individual’s medical history, the age of the injury, and the level of trauma involved [6,7]. Skin is well-known for its limited capacity to accurately record impressions, and it is susceptible to a wide range of potential distortions—an aspect widely acknowledged by bitemark experts. Nevertheless, there is a lack of systematic research studies that have quantified the extent, consistency, correlation, and nature of distortion between dental arches [8]. The biomechanical characteristics of skin dictate that some degree of distortion is inevitable, and this degree varies due to the anisotropic and viscoelastic behavior of skin [9]. Multiple biomechanical properties of skin contribute to this distortion, including nonlinearity and viscoelasticity, which are influenced by the underlying tissues, attachment to the musculature, and anatomical location [10]. Postural distortion manifests when a bitemark is photographed in a position differing from its initial impression during the biting event [11]. The determination of the aging process of bitemarks remains a matter of contention, and there are no universally acknowledged guidelines that provide precise predictions for this intricate phenomenon. Bitemark analysis depends on a combination of morphological and positional information. Geometric morphometric analyses of both 2D [12] and, notably, 3D [13,14] images of dental casts have provided support for the distinctiveness of individual anterior teeth. In recent years, inquiries have arisen regarding the uniqueness of individual dentitions [15]. The morphology of human anterior teeth is related to the teeth of primates in the Anthropoidea taxonomic group [16,17]. Typically, bitemarks on the skin feature two arches that face each other, often corresponding to the anterior maxillary and mandibular dentitions [7]. Instances of wrongful convictions resulting from the misinterpretation of forensic bitemark evidence [18] prompted the American National Academy of Sciences to advocate for scientific research aimed at confirming the uniqueness of human dentition [19]. Guidelines for bitemark analysis, as published by the American Board of Forensic Odontology, emphasize the significance of integrating statistical analyses to enhance the reliability of conclusions [20]. This recommendation is also echoed by the International Organization for Forensic Odonto-Stomatology [21]. Consequently, it is crucial to utilize a probabilistic approach in quantitative analysis methods, ensuring that the evidence presented in court is well supported [22]. The findings of studies examining the uniqueness of human dentition in the context of bitemark analysis [22,23,24,25] have exhibited variations depending on the methodology utilized and the sample’s size [3,26]. Nevertheless, there has been a significant increase in research concerning bitemark identification in recent years, motivated by the aim of achieving quantitative, objective, reproducible, and precise results [5,12,22,23,25,27].

The objective of this review is to investigate the available literature systematically and evaluate the scientific evidence published over the past decade concerning the potential application of bitemark analysis in forensic identification.

## 2. Materials and Methods

### 2.1. Design

This systematic literature review focuses on assessing the potential utility of bitemark analysis in forensic identification. This review adhered to the PRISMA statement (Preferred Reporting Items for Systematic Reviews and Meta-Analyses) guidelines as outlined by Page et al. (2021) [28].

### 2.2. Eligibility Criteria

Strict inclusion and exclusion criteria were applied.

#### 2.2.1. Types of Outcome Measures

The research team created data collection tables to systematically record crucial details from each paper, including sample size, ethnicity, gender distribution, the number of males and females, mean age (including the range), the measurement variables used, and the specific software utilized.

#### 2.2.2. Study Design

This review exclusively included full-text original articles on human bitemarks available in the English language. Experimental studies that satisfied the systematic review criteria were also incorporated, and no limitations were imposed on the age, gender, or ethnic composition of the subjects. The follow-up period was not a factor under consideration.

#### 2.2.3. Inclusion Criteria

To be included, articles needed to meet the following criteria: They had to be full-text original articles focused on human bitemarks, published within the last ten years, and based on experimental or clinical studies involving human subjects. Additionally, the articles had to be available in the English language.

#### 2.2.4. Exclusion Criteria

Articles were excluded if they fell under any of the following criteria: Personal opinions, author debates, letters to the editor, author responses, papers from news groups, abstracts, summaries by editors, congress abstracts, overview papers, and books or book chapters. Furthermore, articles referring to animal experiments, non-English papers, case reports, methodologically inconsistent studies, and systematic reviews were also excluded.

### 2.3. Information Sources

The researchers conducted a thorough search on the Scopus, PubMed, Web of Science, and Cochrane Library databases.

### 2.4. Search Strategy

The researchers conducted a comprehensive search of electronic databases from January 2012 to December 2023, including Scopus, PubMed, Web of Science, and Cochrane Library. Two observers, A.M. and N.C., performed this systematic search using appropriate medical subject headings (MeSHs) and free-text synonyms. Search queries were constructed using Boolean operators such as “AND” and “OR”, along with terms like “bitemark*”, “human bites”, “forensic”, “forensic identification”, and “forensic identification techniques”, in various combinations.

### 2.5. Study Selection

The online literature search was limited to articles published in the English language during the specified timeframe. Importantly, no alterations occurred during the selection and exclusion process, and articles were obtained following strict criteria. Duplicates within the collected studies from these databases were excluded by hand. The process of selecting studies was divided into two phases. In the initial phase, two reviewers (A.M., N.C.) meticulously and independently reviewed the titles and abstracts extracted from all electronic databases to identify articles potentially contributing to the role of bitemarks in forensic identification. In cases where there was disagreement regarding which articles to proceed with for full-text examination, consensus was achieved through discussion. If required, a final decision was reached after consulting with a third reviewer (A.R.). In the second phase, full-text articles were individually assessed for inclusion by two reviewers (A.M., N.C.). Any discrepancies concerning the inclusion of full-text articles were resolved through discussion and, if necessary, by consulting with the third reviewer (A.R.) until a consensus was reached. The reasons for rejecting each paper were documented separately.

### 2.6. Data Collection and Data Items

The same two reviewers, A.M. and N.C., independently gathered data using a predefined and customized data extraction table. Extracted data were cross-checked, and in instances of discrepancies, a consensus was reached through discussion and a re-evaluation of the studies. The data extraction form encompassed the following categories: A. General information (authors’ names, publication year, journal, and ethnicity). B. Key information from each article (measurement types conducted and software utilized). C. Participant details (sample size, gender distribution, and age). The extraction of data was conducted separately and in duplicate by the same two authors, A.M. and N.C., for all the articles ultimately included in the study. Any disagreements arising during the data collection process were resolved through discussion with the involvement of a third author.

### 2.7. Risk of Bias Assessment in Included Studies

The assessment of the included studies’ quality was conducted separately by two reviewers, A.M. and N.C., using the risk of bias in non-randomized studies of interventions (ROBINS-I) tool, which is designed for non-randomized trials and was outlined by Sterne et al. (2016) [29]. The potential for bias in each study was initially assessed individually and later verified by two of the authors. When disparities in evaluation occurred, the authors held detailed discussions to establish a consensus. If no agreement could be reached between the two authors, the article was referred to a third author, A.R., for the ultimate evaluation of quality ratings.

### 2.8. Effect Measures and Data Synthesis

The aim of this project was to investigate the available literature systematically and evaluate the scientific evidence published over the past decade concerning the potential application of bitemark analysis in forensic identification. Author and publication year, sample characteristics (size, gender, and mean age), ethnicity, measurement variables, and software used were the variables identified in each article.

## 3. Results

### 3.1. Description of Studies

**Figure 1:**
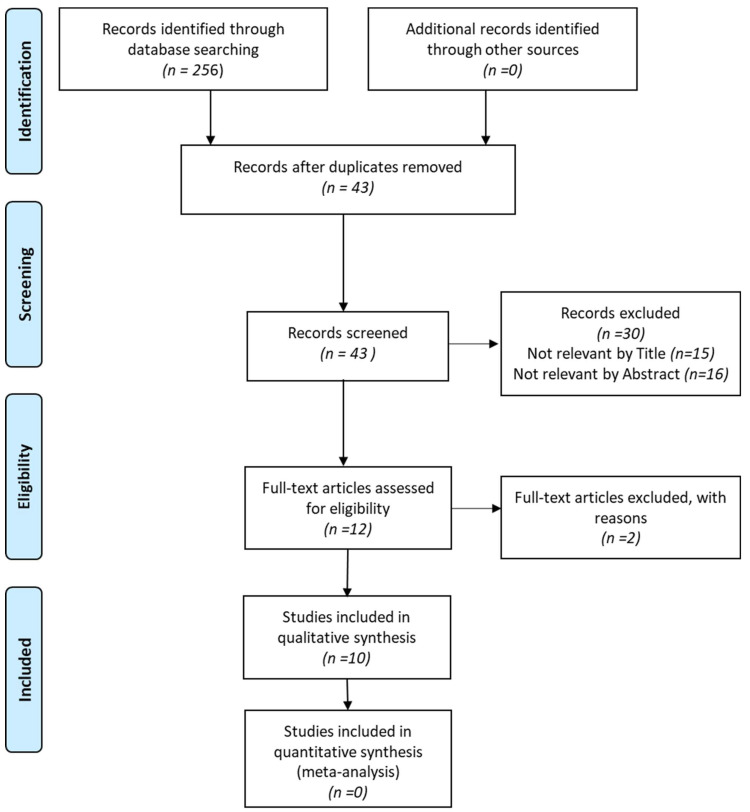
PRISMA flow diagram.

The outcomes of the literature search, including the identification, inclusion, and exclusion of articles, are visually depicted in the flow diagram in accordance with the PRISMA statement (Figure 1). Initially, a total of 256 pertinent records were identified through electronic and manual searches, and after a thorough manual duplicate review, 43 records were retained. Following the screening of titles and abstracts, ten articles met the inclusion and exclusion criteria and were subsequently selected for a comprehensive full review.

The selected articles spanned publication dates from 2012 to 2023. Among the ten identified articles, two presented data from Spanish and Belgian populations, one presented data from France, one from North America (USA), one from India, one from Portugal, one from the United Kingdom, one from Brazil, and one from populations in New Zealand and Scotland. In total, these studies comprised 935 participants, with 296 males and 436 females. In 203 cases, the gender was not reported, as indicated in Table 1.

The sample size in the identified articles exhibited a wide range, with the most limited study involving only 1 volunteer [30], while the most extensive study involved 360 individuals [31]. Among the ten articles reviewed, the majority assessed samples of fewer than 50 participants. However, three studies by Franco et al. (2017), Osborne et al. (2014), and Molina et al. (2020) reported results based on larger participant numbers, specifically, 171, 360, and 65 participants, respectively [31,32,33]. Due to the significant heterogeneity among these articles, a meta-analysis study was not feasible.

**Table 1:** Characteristics of included studies in the systematic review.

Authors Year of Publication	Article Title	Sample Size	Ethnicity	Journal	Gender	Mean Age	Measurement Variable(s)	Software
Martin de-las- Heras et al., 2014 [13]	A quantitative method for comparing human dentition with tooth marks using three-dimensional technology and geometric morphometric analysis	*n* = 13 (10 adults, 3 children)	Spain	Acta Odonto- logica Scandi- Navica	Not mentioned	Not Mentioned (range: 17–65 years (adults), 6–9 years (children)	- 4 (four) incisal angles, - ICM (inter- anine distance	- DentalPrint, - Dig.v 2.10 morphometric
Fournier et al., 2019 [34]	Three-dimensional analysis of bitemarks using an intraoral scanner	8 volunteers	France	Forensic Science International	Not mentioned	Not mentioned	Comparison Dimensions of 3D scans via CloudCompare software (Dentitions & Bitemarks)	- Planmeca Romexis (version 5.1.0.R) - CloudCompare (version 2.9.1) - 3D modelling Software MeshMixer (version 3.4.35)
Sheets et al., 2012 [30]	Bitemarks: Distortion and covariation of the maxillary and mandibular dentition as impressed in human skin	1 volunteer (An apparatus was used to inflict 49 bites on human cadavers sample population of 297 paired maxillary and mandibular dental models	USA	Forensic Science International	Not mentioned	Not mentioned	Landmarks’ comparisons of 297 paired maxillary and mandibular bitemarks	IMP freeware
Osborne et al., 2013 [31]	Does contextual information bias bitemark comparisons?	“Dental” sample *n* = 178 participants “Non-dental” sample *n* = 182 participants	New Zealand	Science and Justice	68 M: 110 F (dental sample) 60 M 122 F (non dental sample)	22.4 years (dental sample), 20.3 years (non-dental Sample)	Evaluation of participants’ capacityto perform more correct matches of bitemark analysis	Not mentioned
Molina et al., 2022 [33]	Dental parameter quantification with semiautomatized computational technology for the analysis of human bitemarks	- 65 dental casts (61 from patients of the School Dentistry clinic and 4 from suspect biters in court cases) - 18 photographs of Bitemarks (2 from victims of court cases and 16 experimental bitemarks in piglet skin).	Spain	Austaralian Journal of Forensic Sciences	Not mentioned	Not mentioned	Evaluation of 5 Parameters (Distance to the arch, Angular position, Eccentricity, Rotation, Intercanine Distance) by a semi-automatized software	- DentalPrint software (University of Granada, Spain, 2004) - Biteprint software (University of Granada, Spain, 2018)
Corte- Real et al., 2018 [35]	Tri-dimensional pattern analysis of foodstuff bitemarks—A pilot study of tomographic database	12 participants	Portugal	Forensic Science International	Not mentioned	Not mentioned	Superimposition of 3D reconstructions of both bitemarks- indicidual’s dental arches, obtained from CBCT database	InVivo 5 Software Anatomage
Reesu And Brown 2016 [1]	Inconsistency in opinions of forensic odontologists when considering bite mark evidence	23 participants	United Kingdom	Forensic Science International	Not mentioned	Not mentioned	assessment consistency of FO opinions, on 4 cases per member(visually comparison of photographs)	Not mentioned
Franco et al., 2017 [3]	Uniqueness of the anterior dentition three-dimensionally assessed for forensic bitemark analysis	171 participants (445 dental casts)	Belgium Brazil	Journal of Forensic and Legal Medicine	81 males: 90 females	Not mentioned	Assessment of statistical significance of mean Euclidean distance (variable compised of 4 components)	Geomagic Studio software
Dama et al., 2020 [36]	Exploring the degrees of distortion in simulated human bite marks	30 anonymised students	Scotland	International Journal of Legal Medicine	6 males: 24 females	Not mentioned (Range 20–50 years)	Exploration of distortion’s degree between a ‘touch mark’ (method 1) and a ‘bite mark’ (method 2) at three different positions’s arm - 6 metric Measurements (teeth #11 and #41: mesio-distal width/ angle rotation and inter-canine distance of upper/lower arch)	Not mentioned
Tarvadi et al., 2016 [37]	Bite Marks Analysis Using Metric Method	50 volunteers	India	Indian Journal of Forensic Medicine & Toxicology	Not mentioned	Not mentioned	- mesio-distal width of each tooth, - intercanine distance	Microsoft Excel Software

Among the ten identified articles, three of them specifically assessed the 3D superimposition of bitemarks [33,34,35]. Additionally, two articles were evaluated using 3D comparisons of bitemarks, combined with linear parameters [13,32]. Two of the articles exclusively focused on evaluating distortion [30,36], while the risk of bias in bitemark comparison was evaluated by two articles [1,31]. Lastly, Tarvadi et al. (2016) examined only linear parameters in their study [37].

Approximately, two-thirds of the articles (6 out of 10) concluded that bitemark analysis was useful in forensic identification [13,30,33,34,35,36,37]. In contrast, four of the identified articles did not report statistically significant results and asserted that the evaluation of bitemarks should not be solely depended upon as a reliable method in the identification process [1,3,31,36].

It is noteworthy that three-dimensional data were collected using a variety of software programs, including CloudCompare version 2.9.1, Planmeca Romexis (Version 5.1.0.R), InVivo5 Anatomage, and IMP freeware.

### 3.2. Risk of Bias

The risk of bias has been assessed for the ten studies included in the final evaluation, as outlined in Table 2. Concerning the overall risk of bias, all of them were categorized as having either a serious or moderate risk. A notable methodological challenge observed in most of the included studies was a lack of a standardized measurement protocols for bitemark landmarks and a failure to include an assessment for potential distortion.

## 4. Discussion

In this comprehensive review, an extensive examination of the prior literature concerning the role of bitemark analysis in forensic identification was conducted, utilizing data from four distinct electronic databases. The fundamental principle of bitemark analysis is the belief that the dental characteristics of the anterior teeth involved in biting are distinctive among individuals, and it is assumed that this distinctiveness is transferred and imprinted in the injury [38,39]. However, it is important to note that Bush et al. (2009) conducted a study where they replicated human bites on a human cadaver and examined the resulting skin marks. Their findings suggested that bitemarks created by similar dentitions could not be consistently differentiated [10]. Consequently, further research is necessary to definitively establish the uniqueness of each individual’s dentition [40]. Conversely, a different study involving digitized three-dimensional bitemarks reported that 15% of the combinations of dentitions and bitemarks were incorrectly identified as matches [23]. This highlights the intricate and ongoing debate regarding the reliability of bitemark analysis in forensic identification.

Landmark-based geometric morphometric (GM) analysis is a widely recognized method for characterizing size and shape variations in biological specimens. In GM, landmarks are strategically positioned on digital images of the specimens. These landmarks are recorded as coordinates, effectively capturing and preserving spatial information. Subsequently, these landmarks can be extracted and employed to quantitatively and statistically articulate shape variations among specimens. This is achieved through a range of multivariate statistical techniques [41]. The analysis of bitemarks should be approached cautiously and is best utilized as a supplementary method, primarily for excluding suspects rather than confirming a definitive match. Similar conclusions were reached in the studies conducted by Fournier et al. (2019), Tarvadi et al. (2016), and Sheets et al. (2012) [30,34,37].

Fournier et al. (2019) conducted a study to assess the reliability of their 3D analysis protocol for bitemarks using an intraoral camera and mesh comparison software [34]. Their preliminary investigation involved eight volunteers, from whom they obtained eight whole dentitions (comprising eight maxillary and eight mandibular arches). Dental impressions were taken using alginate, and stone casts were created with a dental type III stone material. In their experiment, each volunteer bit into three different materials, dental wax (Material 1), a false forearm covered with a dental wax layer (Material 2), and a hard cheese, to create an avulsive bite (Material 3). Consequently, they generated 24 distinct bitemarks and eight pairs of stone models. The dentitions and bitemarks were then scanned using an intraoral digital scanner called PlanMeca Emerald, along with Romexis software (Version 5.1.0.R) for digital acquisition and 3D prosthetic design. Landmarks were selected at the middle of the incisor edges, and CloudCompare software (Version 2.9.1)was used to calculate distances to the nearest points. This software provided data on the minimal distance, maximal distance, mean distance, and standard deviation, and color-coded histograms and overlays showed color variations in the bitemark images. Two types of comparisons were conducted: a visual assessment and an isolation of indentations from the surrounding material, with a focus on comparing the dental edges to these isolated indentations, which was specifically done for bitemarks on wax. Visual observations quickly and easily excluded false matches, and all the bitemarks were successfully matched to their corresponding dentitions. Despite this 100% success rate, the authors emphasized that their method was more suitable for excluding suspects in forensic cases than for confirming perfect matches [34].

Their proposed 3D bitemark analysis protocol was not only quick (taking approximately 20 min) but also aligned with the American Board of Forensic Odontology (ABFO) guidelines revised in 2018 [20]. These guidelines specify three possible outcomes at the end of bitemark analysis: the dentition can be excluded as the source of the bitemark; the dentition cannot be excluded (referred to as inclusion); or the information is insufficient for a conclusive determination. Thus, forensic odontologists cannot definitively confirm a perfect match but can only exclude or suggest probable inclusion [20]. Nevertheless, their study has several limitations. The colorimetric scale they adapted might face criticism, since the green coloration could be susceptible to the inherent errors of the camera. Additionally, the eight complete dentitions they utilized had some individual characteristics, such as tooth rotations or translations, which could potentially facilitate the exclusion of a dentition in specific cases. Furthermore, PlanMeca intraoral scanners did not capture real colors, but rather approximations, which could affect the accuracy of bitemark analysis on skin.

Tarvadi et al. (2016) conducted a study aimed at assessing the applicability of bitemark analysis for forensic purposes, specifically using a metric method [37]. They recruited a sample of 50 volunteers who were instructed to bite their own forearms. From the resulting negative impressions, the researchers utilized vernier calipers to measure 14 linear parameters. These parameters included the mesio-distal width of each anterior tooth (six maxillary and six mandibular) and two inter-canine distances. Subsequently, all 50 bitemark casts were individually compared to the dentitions of all 50 volunteers. Among the 50 cross-matches, the outcomes revealed 14 true positives and 36 false positives, indicating a substantial error rate of 72%. The authors concluded that this method of bitemark analysis was more suited for excluding suspects rather than providing definitive positive identifications [37].

Nonetheless, it is crucial to acknowledge several limitations within their study. Firstly, they did not specify the precise positioning of the vernier calipers, potentially introducing subjectivity and impeding the replicability of measurements. Furthermore, they did not clarify whether they utilized univariate or multivariate statistical analysis, and they did not provide any results tables or graphs. Their argument heavily relied on manual measurements performed by a sole individual, raising the potential bias. Sheets et al. (2012) performed a study with the objective of assessing arch width distortion and the covariation of both maxillary and mandibular dentitions [30]. To achieve this, they obtained polyvinylsiloxane impressions of the maxillary and mandibular arches from a single volunteer. Using a single dentition (either maxilla or mandible), they created 49 bitemarks on unembalmed cadavers. These resulting bitemarks were digitally photographed, and landmarks were placed on digital images of the bitemarks as well as scanned images of the biting dentition.

For comparison purposes, they utilized a sample of 297 dental models that were randomly acquired. Although they conducted 10 intra-examiner repeat measurements on 3 randomly chosen images of bitemark specimens and 3 scanned dental images, achieving a high level of agreement between them, the outcomes revealed a poor correlation between the arch widths of the maxilla and mandible in the bitemarks produced by the single dentition. The limited R-squared value for both upper and lower arches (approximately 0.3) indicated significant independent variation in arch width between the maxilla and mandible. The variation in bitemark arch width was found to be 7–28 times larger than the measurement error in the dentition and roughly 50% of the variation observed in the clinical population of dentitions. Furthermore, all the bitemarks in this study exhibited some degree of distortion, and this distortion exceeded the measurement error range of the dentition [30]. The authors acknowledged several limitations of their study. Firstly, the bitemarks were generated on cadavers, potentially lacking fidelity to what would be encountered in living tissue. Additionally, all the bites were produced utilizing a single dentition, leaving uncertainty regarding the generalizability of the study’s findings to other dentitions.

In the pilot study by Corte-Real et al. (2018), the authors aimed to develop a reliable and accurate protocol for digital three-dimensional (3D) analysis to enhance the consistency of bitemark analysis as forensic evidence [35]. To accomplish this, they selected 12 cranium Cone Beam Computed Tomographic (CBCT) files randomly from a clinical database (Coimbra Hospital and University Center/Faculty of Medicine of the University of Coimbra) based on specific inclusion–exclusion criteria. Subsequently, the participants in the study were instructed to bite an apple and then immediately underwent a CBCT scan.

The 3D renderings of each bitemark were compared with the 3D upper dental arches obtained from the CBCT cranium scans of the simulated participants. This involved superimposing 12 upper dental arches with 12 bitten apples, resulting in a total of 144 scenarios or comparisons for analysis. To ensure the reliability and consistency of the outcomes, a research team comprising five members underwent training and calibration in the utilization of the software. The outcomes were classified into four distinct rating scales: (1) match (all interdental incisor contact points aligned); (2) probable match (two or more interdental incisor contact points coincided); (3) probable mismatch (indicating one interdental incisor contact point match); and (4) mismatch (none of the interdental incisor contact points matched). The matching procedure used comparable landmark spots in both the bitemark and the participants’ maxillary teeth. Given that the normality assumption was not satisfied, a non-parametric test (Friedman’s test) was utilized. The outcome of the Friedman’s test lacked sufficient power to reject the null hypothesis (H_0_ = the group medians were all equal), leading to the assumption that the uniqueness of human dentition (and consequently, bitemarks) did not exist. The authors introduced an original and non-destructive study design for a bitemark pattern analysis protocol. This approach entailed a direct comparison between the scan of the bitten foodstuff with the scan of the cranium of the participant. This procedure was not dependent on the operator, which addressed one of the main limitations and biases associated with manual landmarking techniques [35]. According to the study conducted by Reesu and Brown (2016), it is important to approach bitemark evidence with caution [1]. Their research revealed inconsistencies in opinions among forensic odontologists, which varied in whether a bitemark could be attributed to a human or an animal, as well as in whether it was inflicted by an adult or a child. In their survey, 23 forensic odontologists participated and were asked to complete a questionnaire that included four photographs of bitemark cases for research purposes. The findings of the study highlighted discrepancies in perspectives among the odontologists [1].

Furthermore, there were conflicting views on determining whether an adult or a child was responsible for causing the bitemark. Even experienced forensic odontologists found it challenging to assess bitemark evidence, and the degree of certainty in their opinions (ranging from definite to probable to possible) varied among experts. Notably, in the study by Reesu and Brown (2016), even very experienced forensic odontologists altered their conclusions 50% of the time. Interestingly, this rate of revision was lower than that observed among both novices and recently educated MSc. students, emphasizing the complexity and subjectivity of bitemark analysis [1]. These findings align with previous research. Both workshops conducted by the American Board of Forensic Odontology (ABFO) and studies conducted in Australia have also identified discrepancies and varying opinions among individuals when assessing bitemark cases [28,42]. Additionally, Bush et al. (2009) conducted a study in which they simulated human bites on a human cadaver and analyzed the resulting marks on the skin. Their research demonstrated that bitemarks created by similar dentitions could not be consistently distinguished [10]. In the study conducted by Franco et al. (2017), the researchers aimed to assess the potential uniqueness of the human dentition (UHD) in a three-dimensional (3D) context [32]. They investigated this distinctiveness using different quantities of dental material from the incisal edges. The uniqueness of human dentition was considered to have been established when the mean Euclidean distance within any studied group was statistically significantly greater than the corresponding reference group. To perform their analysis, the researchers collected 445 dental casts, which were used to create four study groups: (I) randomly selected subjects, (II) orthodontically treated subjects, (III) twins, and (IV) orthodontically treated twins. Furthermore, 20 dental casts were used to generate threshold groups of people from whose dental impressions were collected at two distinct times (Group V). [32]. The researchers compared the four study groups with their respective threshold groups using an ANOVA test, with a statistical significance level of 5%. They found that Groups I, II, and III did not exhibit statistically significant differences from their respective thresholds (Group V) in all aspects of the study (*p* > 0.05). This lack of statistical significance led to the conclusion that scientific evidence to support the concept of UHD was not observed in their study. They also noted that, in forensic practice, investigations involving bitemarks and the use of simulated standards can only be reliably performed in closed populations [3]. However, it is important to acknowledge several limitations in their study. One limitation was the absence of mandibular dental cast files from orthodontically treated subjects, and the authors encouraged the use of this material in future studies in the field. Another limitation pertained to the possibility of errors inherent in the manual process of taking dental impressions and producing dental casts in plaster, which can be influenced by the operator’s skill. To address this limitation, the potential error during the manual procedure was incorporated into the threshold group [3].

In the study conducted by Dama et al. (2020), the researchers aimed to investigate the degree of skin distortion between a “touch mark” and a simulated “bite mark” on the middle third area of the left upper arm at three different positions [36]. They used a Nikon DX digital camera (D5000) to capture photographs from a sample consisting of 30 subjects (6 males and 24 females) aged between 20 and 50 years old. The study identified significant statistical differences in the “mark types” and their positions. When analyzing the results of “bite marks,” a significant degree of distortion was detected. Conversely, when examining the results of “touch marks” for the upper arch, there was no statistically significant difference in the mesiodistal (MD) width of tooth #11 and the inter-canine distance at all positions. However, a significant degree of distortion was observed in the angle of rotation of tooth #11 at all positions. Similarly, when analyzing the results of “touch marks” for the lower arch, there was no statistically significant difference in MD width and the angle of rotation of tooth #41 and the inter-canine distance at all positions.

These findings underscored the influence of skin properties and posture on distortion. Such distortion could potentially lead to inaccurate measurements and a misleading interpretation of bitemark injuries [36]. These results were consistent with prior studies on bitemarks on human skin, emphasizing how the biomechanical properties of the skin and changes in body posture contribute to the observed distortion in bitemarks [43,44]. The authors suggested that the angulation of the tooth mark may be altered due to skin properties and posture, casting doubt on angulation as a strong dental characteristic for identification. Furthermore, the study highlighted that the degree of distortion varied in bitemarks, affecting arch size and shape [11]. It is important to note that inter-canine distance has been used in forensic analysis to differentiate the origin of marks (human or animal), distinguish between human adult bites (deciduous teeth of adults, small adults, or children), and even estimate specific race and sex groupings due to its relevance [45,46].

The study by Osborne et al. (2014) was designed to investigate the presence of contextual bias in bitemark analysis. Participants were recruited from two different populations: dental students and non-dental students [31]. The dental sample comprised 178 undergraduate students enrolled in the School of Dentistry at the University of Otago, New Zealand. The non-dental sample consisted of 182 undergraduate Psychology students, also from the same university. Bitemark impressions were collected from 15 volunteers who produced clear impressions of maxillary orthodontic dental casts on the skin. These impressions were then photographed using a Cannon Powershot G11 camera. The study’s findings revealed that the provision of contextual information influenced participants’ decisions when assessing ambiguous bitemarks. Interestingly, when participants were presented with highly emotional images and subliminally primed with the word ‘guilty’, they made fewer matches compared to the control condition. Furthermore, dental expertise influenced decision making, as dental students made more matches as the trial went on, independent of the context or task ambiguity [31]. In Molina et al.’s (2020) study, the primary objective was to identify the specific quantitative dental parameters that characterized human bitemarks and dentitions. This analysis was intended to enhance the credibility of bitemark analysis in forensic cases, addressing the courts’ demand for quantitative statistics rather than purely descriptive analysis. To accomplish this objective, the researchers employed a semi-automated technique for calculating various parameters. These parameters encompassed the inter-canine distance, rotation, eccentricity, angular position, and distance to the arch for each tooth mark. This approach allowed them to compare bitemark photographs with 3D images of dental casts, reducing the subjective element in human bitemark analysis. Among these parameters, the rotation of lower teeth emerged as the most accurate in identifying the biter. The results indicated that this procedure could potentially be employed in criminal trials involving human bitemarks [33].

However, it is crucial to acknowledge certain limitations in Molina et al.’s (2020) study. Bitemark indentations with sufficient discriminative power for use in forensic cases are rarely found on victims’ skin. Consequently, the analysis in this study focused on bruising resulting from tooth marks, which, as they healed, can undergo diffusion and potential movement. Additionally, most bitemarks were created on the skin of freshly slaughtered piglets, which do not faithfully replicate the viscoelastic properties of human skin, the exact location of bite injuries, or skin movement. Another limitation was that the semi-automatic BitePrint procedure still involved expert input in two steps: drawing the initial ellipse and labeling the tooth types in the bitemark. Furthermore, capturing tooth marks with 2D technology may be less accurate than using 3D technology, potentially affecting subsequent bitemark analysis.

In Martin-de-las-Heras et al.’s (2014) study, they aimed to evaluate specific dental parameters by comparing 3D overlays created from dental casts with experimental bitemarks. To accomplish this, the authors used thirteen upper and lower dental casts, which were 3D-scanned to generate comparison overlays using DentalPrint (Dig.v 2.10) software. Their analysis encompassed five measurement variables, comprising four incisal angles and the inter-canine distance. The outcomes demonstrated that all single (angle or distance) and combined (logistic model) variables had statistically significant discriminative power. The lower 95% confidence interval (CI) limits for the areas under the ROC (Receiver Operating Characteristic) curves were greater than 0.50, indicating good discriminatory ability. Sensitivity and specificity values were also greater than 50% for both the maxilla and mandible [13].When it came to matching, the most accurate variable was the maxillary inter-canine distance (ICD) with a small discrepancy of 1.8 ± 2.2%. The angle of tooth 32 also showed a relatively high accuracy with a discrepancy of 3.1 ± 2.2%. However, the angle of tooth 42 had the least favorable results, with a discrepancy in matching reaching 15.6 ± 29.2%. Applying strict statistical interpretation, the maxillary ICD proved to be highly accurate for the identification procedure, with an area under the ROC curve of 0.9 ± 0.1 and a lower 95% CI limit of 0.9. Furthermore, the difference between matching and non-matching incisal angle values was smaller in the mandible compared to the maxilla, suggesting that incisor rotation exhibited less individuality in the mandible [13]. Geometric morphometric (GM) analysis has also been applied to bitemarks to describe and compare their shapes [26,27]. This approach quantitatively assessed shapes by capturing the geometric characteristics of relevant morphological structures. The advent of advanced 3D bitemark digital imaging has enabled precise calculations of diverse dental parameters using GM analysis. These parameters encompassed inter-canine widths, mesial-distal lengths, rotations, variations in tooth height, and other relevant variables [13]. On the contrary, some other researchers have observed a more significant disparity between mandibular and maxillary dentitions, attributing it to a higher prevalence of crowding in the lower arch [25]. Nevertheless, it is essential to recognize that their findings might not be directly applicable to bite injuries. A prior study that used 3D technology for the quantitative assessment of human dentitions using generated bitemarks revealed sensitivity and specificity scores of 78% and 85%, respectively [23]. Furthermore, an investigation focused on experimental bitemarks aimed to quantify the probability of a specific match. However, this study utilized 2D images of a 3D model, and the authors acknowledged the need for a more realistic set of bitemarks [22].

This systematic review had several limitations to consider. A substantial portion of the examined articles did not report statistically significant results, which raises questions about the reliability of some of the findings. Moreover, a considerable number of the included articles were characterized by a serious risk of bias, indicating potential methodological issues that could impact the validity of the results. All measurements and evaluations were conducted immediately after the bitemarks’ creation. It is unclear whether the effectiveness of identification would change if a longer period elapsed, which could be an important consideration in forensic cases. Additionally, the final dimensions of the bitemark may have been influenced by the specific objectives of each study. This implies that the results might not be directly applicable to all forensic scenarios. For instance, in the study by Tarvadi et al. (2016), the absence of a published table and the lack of statistical analysis or detailed data processing information pose challenges in assessing the study’s rigor and reliability [37]. Some of the measurement methods used in the reviewed articles were subjective, introducing potential bias, reducing the reliability of the findings. The intervention status in the reviewed studies was not well defined, further complicating the understanding of the precise procedures and methodologies employed. These limitations highlighted the need for more standardized and rigorous research in the field of bitemark analysis to ensure its reliability and accuracy in forensic identification.

Research in the field of bitemark analysis has illuminated a range of challenges and limitations that warrant attention [47]. The forensic examination of patterned injuries, such as bitemarks, is significantly affected by distortion, which can be classified into primary and secondary forms. This distortion complicates the precise analysis of such injuries [11]. Despite suspicions surrounding patterned injuries like human bitemarks, many cases never progress beyond the initial analysis stage [48]. Studies have disclosed error rates in matching dentitions with bitemarks. For instance, research involving digitized three-dimensional bitemarks revealed a 15% rate of incorrect identifications, while similar investigations on bitemarks in pig skin reported error rates ranging from 11.9% to 22% [23,40]. The validity of bitemark evidence in court was officially questioned in 2009, prompting intensified scientific exploration in three primary domains: assessing the uniqueness of human dentition (UHD), comprehending the distortion of bitemarks on skin, and refining the technical interpretation of bitemark evidence [19]. Research on UHD has unveiled noteworthy variability in the methodology. Studies have employed 2D- and 3D-image registration, dental casts or wax indentations, and diverse approaches to scrutinize the contour or morphology of incisal edges [5]. These findings underscored the imperative need for standardized methodologies, rigorous research practices, and enhanced techniques in bitemark analysis to augment its reliability and accuracy in forensic inquiries.

Nevertheless, there exist several domains where enhancements in bitemark analysis can be pursued. Bitemark analysis should be applied judiciously, primarily in closed populations featuring two suspects with distinguishable dentitions. This approach holds the potential to heighten the precision of bitemark analysis [3]. It is imperative to confine bitemark analysis to cases of high forensic significance. Such cases facilitate the measurement of dental parameters, enabling the generation of scientific data concerning the probability of a match between a toothmark and a dentition [49]. The introduction of DNA evidence has revealed inaccuracies in certain bitemark reports. This highlights the need for complementary and more reliable forensic techniques [49,50]. Although 3D bitemarks (indentations) can offer remarkable precision for identification purposes, they often disappear or are altered over time due to the viscoelastic properties of the skin, leaving only hematomas. This limitation underscores the necessity to develop quantitative and less subjective techniques [51]. These proposed improvements aim to enhance the reliability and scientific validity of bitemark analysis in forensic investigations. The field of bitemark analysis has encountered substantial criticism and challenges in recent years. The 2009 report from the National Academy of Sciences (NAS) highlighted numerous concerns, including the lack of scientific validation, error rate determination, and reliability testing in bitemark analysis. It also emphasized the absence of fundamental scientific research in this field [19,40]. The International Organization for Forensic Odonto-Stomatology (IOFOS) has similarly recommended that expert conclusions in this area should be based on scientific probabilistic studies [21]. Recognizing the three-dimensional nature of bitemark injuries, many experts advocate for approaches based on 3D technology [52]. The adoption of 3D methodologies has demonstrated their capacity to improve the precision of human bitemark analysis while diminishing subjective biases. However, it is crucial to note that 3D technology alone cannot address all the challenges associated with bitemark analysis, such as those related to skin distortion. Hence, there remains a need for ongoing scientific research to improve this technique [13].

## 5. Conclusions

Bitemark analysis functions as an adjunctive method with significance in forensic identification. Bitemark analysis can be primarily used for excluding rather than identifying a specific individual; bitemark analysis alone is insufficient for identification; additional procedures must also be employed. This needs to be emphasized since, otherwise, legal personnel may overestimate the reliability of bitemark evidence, resulting in false convictions. Timeliness in addressing recently inflicted bitemarks is crucial, and the establishment of a universally accepted protocol for data collection, processing, and analysis is imperative. Such a protocol should be collaboratively developed and adopted by the worldwide scientific community, offering potential benefits across diverse forensic applications. Numerous scholars concurred that utilizing 3D analysis with specific software represents a more objective methodology in contrast to other approaches. This improves the analytical process and mitigates inherent biases. To enhance the dependability and precision of bitemark analysis, further studies involving larger sample sizes are imperative. Such research endeavors could potentially transform bitemark analysis from a primarily exclusionary technique into one with a broader scope of inclusion-based identification.

## Figures and Tables

**Table 2:** Risk of bias of included non-randomized studies, according to ROBINS-I tool.

Bias Due to/in								
	Confounding	Selection of Participants for the Study	Classification of Interventions	Deviations from Intended Interventions	Missing Data	Measurement of Outcomes	Selection of the Reported Result	Overall
Martin de-las-Heras et al., 2014 [13]	Low	Moderate	Serious	Serious	Moderate	Moderate	Moderate	Serious
Fournier et al., 2019 [34]	Low	Moderate	Moderate	Moderate	Moderate	Serious	Serious	Serious
Sheets et al., 2012 [30]	Moderate	Moderate	Low	Serious	Low	Serious	Low	Serious
Tarvadi et al., 2016 [37]	Moderate	Moderate	Low	Moderate	Low	Serious	Moderate	Serious
Osborne et al., 2013 [31]	Serious	Serious	Low	Serious	Low	Serious	Moderate	Serious
Molina et al., 2022 [33]	Low	Low	Low	Low	Moderate	Moderate	Moderate	Moderate
Corte-Real et al., 2018 [35]	Low	Moderate	Low	Moderate	Low	Moderate	Moderate	Moderate
Reesu and Brown. 2016 [1]	Moderate	Moderate	Low	Moderate	Low	Moderate	Moderate	Moderate
Franco et al., 2017 [3]	Low	Moderate	Low	Moderate	Low	Moderate	Moderate	Moderate
Dama et al., 2020 [36]	Low	Moderate	Moderate	Moderate	Moderate	Moderate	Moderate	Moderate

## Data Availability

No new data were created or analyzed in this study. Data sharing is not applicable to this article.
